# Task-specific facial dystonia following COVID-19 infection: A case report

**DOI:** 10.1097/MD.0000000000039689

**Published:** 2024-09-13

**Authors:** Jong-Mok Lee, Pan-Woo Ko

**Affiliations:** a Department of Neurology, School of Medicine, Kyungpook National University, Daegu, Korea; b Department of Neurology, Kyungpook National University Hospital, Daegu, Korea.

**Keywords:** botulinum, COVID-19, focal dystonia

## Abstract

**Rationale:**

During the coronavirus disease of 2019 (COVID-19) pandemic, various movement disorders associated with COVID-19 infection have been reported. However, the incidence of dystonia specifically associated with COVID-19 infection has been particularly rare.

**Patient concerns:**

A 43-year-old male patient presented to the movement disorders clinic with complaints of facial grimacing while chewing and experiencing spasms during nasal breathing. These symptoms appeared 2 weeks after he tested positive for COVID-19.

**Diagnoses:**

Based on normal diagnostic test results, including brain imaging and blood tests, it was concluded that task-specific facial dystonia following COVID-19 infection.

**Interventions:**

Despite treatment with clonazepam, trihexyphenidyl, and carbamazepine, his condition did not improve. Subsequently, botulinum toxin injections were administered to the affected facial muscles identified through video analysis.

**Outcomes:**

Botulinum toxin injections led to a significant improvement in the patient’s symptoms.

**Lessons:**

Task-specific dystonia affecting the facial muscles, particularly induced by specific actions such as chewing and nasal breathing, is rare and may represent an atypical post-infectious manifestation of COVID-19.

## 1. Introduction

Task-specific dystonia is characterized by persistent motor incoordination triggered by skilled movements or repetitive actions. Its estimated prevalence ranges from 7 to 69 cases per million individuals.^[1,2]^ While musician’s dystonia and writer’s cramp are the most common forms, they can also be induced by various other actions. Diagnosis of task-specific dystonia, similar to different types of dystonia, involves ruling out genetic and secondary causes, and movement disorder specialists use phenomenological criteria.^[3]^ Treatment options vary based on the patient’s symptoms. They may include medications like trihexyphenidyl, primidone, and baclofen, as well as non-pharmacological approaches such as botulinum toxin injections, deep brain stimulation, and physical therapy.^[4]^ Despite ongoing research, the exact cause remains unclear, with hypotheses focusing on abnormalities in inhibition, plasticity, and motor networks.

Coronavirus disease of 2019 (COVID-19), caused by severe acute respiratory syndrome coronavirus 2, primarily induces respiratory symptoms but has also been associated with various neurological symptoms, including headache, dizziness, olfactory dysfunction, and delirium/confusion. Movement disorders such as myoclonus, ataxia, and tremors have also been reported.^[5,6]^ Although cervical and generalized dystonia have been associated with COVID-19 vaccination, focal dystonia has rarely been documented. Specifically, focal task-specific dystonia (FTSD) following COVID-19 infection has not been reported in the literature before. In this case, we present a rare instance of task-specific facial dystonia induced by chewing and breathing following a COVID-19 infection.

## 2. Case presentation

**Figure 1.**
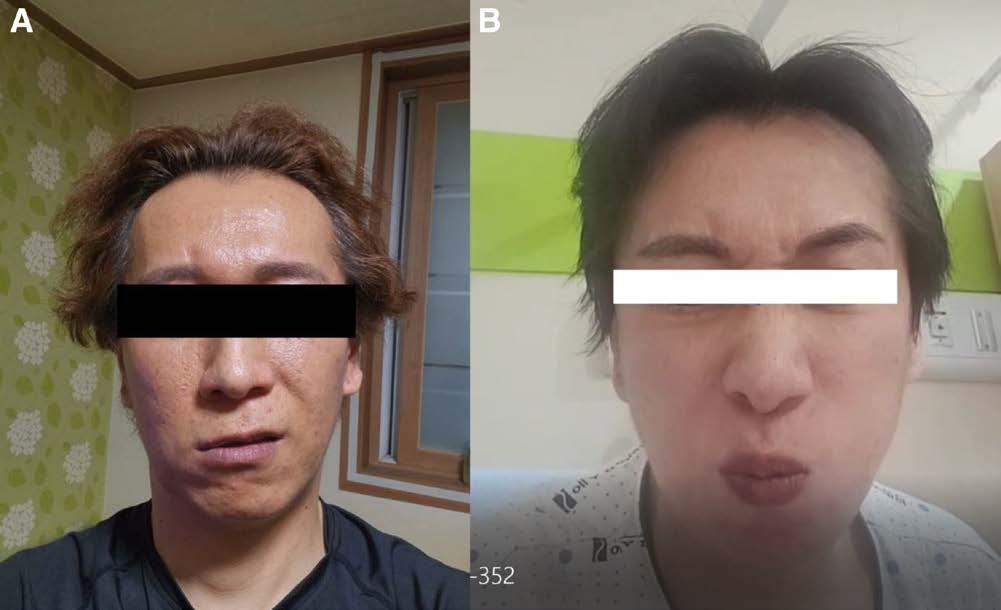
The patient remained asymptomatic when at rest. However, muscle contractions, particularly in the levator labii superioris alaeque nasi, were observed during deep nasal breathing (A). Contractions of the orbicularis oris and procerus muscles were noted during chewing (B).

**Figure 2.**
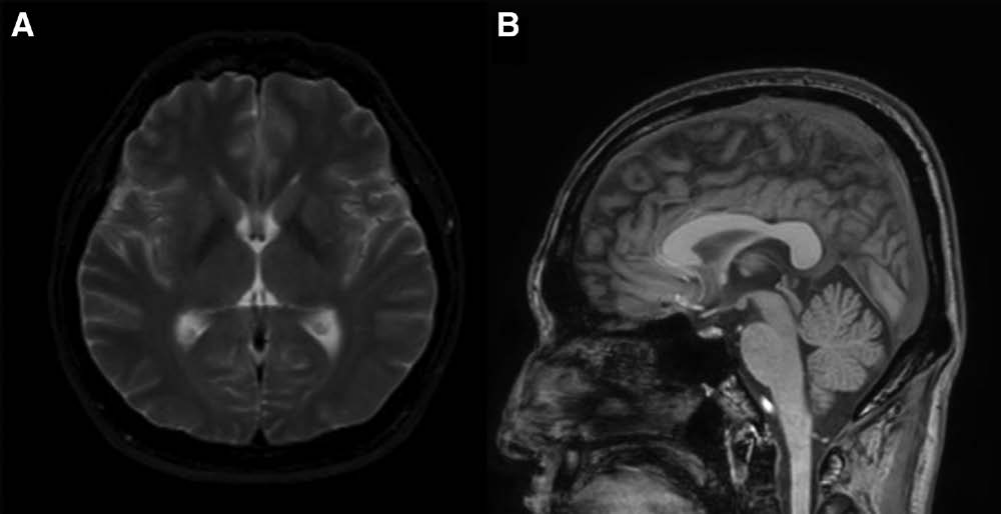
(A) Axial T2-weighted image. (B) Sagittal T1-weighted image. No abnormalities were observed on the patient’s brain MRI. MRI = magnetic resonance imaging.

A 43-year-old man visited the movement disorders clinic due to facial grimacing while eating that had persisted for several months. Six months prior, in August 2022, he had experienced confirmed symptoms of COVID-19, including fever, myalgia, and fatigue. He received conservative treatment and later recovered. However, 2 weeks after the COVID-19 diagnosis, he developed symptoms of facial spasms during nasal breathing (Fig. 1A) and facial grimacing while chewing (Fig. 1B). The patient was admitted to a local neurology hospital to rule out acute secondary causes, including cerebrovascular disease. Diagnostic investigations, including brain imaging were conducted. The results of the brain magnetic resonance imaging were normal (Fig. 2). Nerve conduction studies and blood tests revealed no abnormalities that could explain the symptoms. The patient reported trying trihexyphenidyl, clonazepam, and carbamazepine sequentially for symptom management, but none were effective. After 6 months of evaluation and treatment without symptom improvement, he was referred to a tertiary medical center and visited our clinic. A neurological examination conducted at our clinic revealed no abnormalities in cranial nerve, motor, or sensory functions. Abnormal movements were not observed at rest but only during chewing and nasal breathing. Symptoms were alleviated with sensory tricks such as touching the nose and were absent during mouth breathing. No abnormal movements were observed during swallowing, drinking, speaking, or singing. Video analysis showed the levator anguli oris contraction during nasal breathing and the procerus, nasalis, and levator labii superioris alaeque nasi muscles during chewing (Video 1, Supplemental Digital Content, http://links.lww.com/MD/N582). The patient had no family history of dystonia or previous medication use. There were no significant medical events other than the COVID-19 infection 2 weeks before symptom onset. Since brain imaging and other tests showed no abnormalities, and dystonia was induced by specific actions such as chewing and nasal breathing, he was diagnosed with task-specific facial dystonia following COVID-19 infection. As there was no improvement in symptoms with medication treatment from the previous hospital, and the patient reported significant social discomfort due to dystonia, botulinum toxin injection treatment was planned. Abobotulinumtoxin A was injected into the muscles identified to be contracted during video analysis. Botulinum toxin injection significantly improved symptoms, excluding nasal breathing-induced nostril flaring symptoms (Video 1, Supplemental Digital Content, http://links.lww.com/MD/N582). However, the symptoms recurred after 2 to 3 months; thus, periodic botulinum injection was planned.

## 3. Discussion

Throughout the COVID-19 pandemic, various movement disorders associated with COVID-19 have been reported.^[7]^ Ataxia, myoclonus, parkinsonism, dystonia, and chorea are among the post-COVID-19 movement disorders.^[5]^ However, the incidence of dystonia specifically associated with COVID-19 infection has been particularly rare. A review article on movement disorders related to COVID-19, published in 2023, identified only 5 cases of dystonia out of 133 movement disorder cases analyzed.^[8]^ Similarly, another study reported that out of 81 cases of post-COVID movement disorders, only 3 presented with dystonia symptoms.^[7]^ When cases of dystonia mentioned in the review article and subsequent publications were compiled, excluding duplicate cases, a total of 6 cases of dystonia following COVID-19 infection were confirmed, as shown in Table 1. The interval between COVID-19 infection and the onset of dystonia ranged from several hours to 11 weeks. In 5 cases, the dystonia presented as segmental dystonia affecting the upper limb, shoulder, and neck regions,^[10–13]^ while in 1 case, it manifested as focal dystonia of the facial muscles.^[9]^ Among the 4 cases with described outcomes, 3 cases spontaneously recovered with conservative treatment, and the case with orofacial dystonia showed improvement with botulinum toxin injection therapy. Notably, the reported instances typically involved dystonia in patients with preexisting Parkinson disease or manifested as phasic dystonia of the shoulder or neck. Task-specific dystonia, like the one presented here, has not been reported in the literature.

**Table 1:** Characteristics of patients with dystonia following COVID-19 infection.

Article	Age/gender	Neurological symptoms	Time between infection and onset of neurological symptoms	Treatment/outcome
Girouard and Levy^[9]^	Female/61	Blepharospasm, orofacial dystonia	7 d	Botulinum toxin injection/partial success
Franke et al^[10]^	Male/78	Dystonia of right upper limb	Not informed	Not informed
Lo Monaco et al^[11]^	Female/58	Limb dystonia	Hours	Spontaneous recovery
Zimmermann et al^[12]^	Female/29	Phasic dystonia of the shoulder and trunk	9 d	Spontaneous recovery
Zimmermann et al^[12]^	Male/59	Phasic dystonia of the neck	Not informed	Spontaneous recovery
Morassi et al^[13]^	Female/70	Focal dystonia of the right upper limb	11 wk	Not informed

COVID-19 = coronavirus disease of 2019.

The underlying mechanisms of movement disorders following COVID-19 infection remain unclear. Hypotheses suggest factors such as hypoxia, direct viral invasion, and cytokine-mediated immune responses.^[14]^ Since the pathophysiology of both neurological symptoms related to COVID-19 infection and dystonia remains unclear, elucidating the mechanism by which COVID-19 triggers dystonia presents a challenge. Various hypotheses can be considered. Loss of smell and taste is a well-known complication of COVID-19 infection.^[15]^ Although the precise mechanism remains unclear, hypotheses include direct viral invasion, immune response-related inflammatory edema, and vascular damage affecting neurological function.^[16]^ Dystonia’s pathophysiology involves mechanisms such as loss of inhibition and abnormal plasticity, leading to abnormal sensorimotor integration.^[17]^ Combining these hypotheses, we can infer potential causes for the task-specific dystonia observed in this case. COVID-19 primarily affects the respiratory symptoms, with the virus mainly entering through the nasopharynx. This entry could cause sensory system impairment through direct or immune-mediated effects, disrupting sensorimotor integration and leading to dystonia. The task-specific facial dystonia observed in this case, triggered by nasal breathing or chewing, could be understood as an overflow phenomenon resulting from sensorimotor integration impairment. Similar hypotheses have been proposed for jaw dystonia induced by biting, suggesting that sensory input changes could alter central control, causing abnormal movements.^[18]^

FTSD is characterized by action-induced dystonia affecting isolated body parts, often triggered by specific, repetitive tasks such as writing or playing musical instruments. However, in our case, the patient did not exhibit specific occupational habits or repetitive movements akin to musician’s dystonia or writer’s cramps. Interestingly, the patient displayed dystonic movements during chewing or nasal breathing, accompanied by sensory tricks, suggesting task-specific dystonia. Reports of chewing- and inspiration-induced dystonia are scarce.^[19–21]^ Most patients did not present a history of infection symptoms and exhibited idiopathic movements. Although the specific mechanism and relation to COVID-19 infection, in this case, are challenging to elucidate, the absence of significant medical history and normal results of diagnostic tests, including magnetic resonance imaging, led to the conclusion that COVID-19 infection was the cause of the patient’s symptoms.

A limitation of this case study lies in establishing a clear relationship apart from the temporal association between COVID-19 infection and the manifestation of dystonic movement symptoms. When it comes to focal facial dystonia, differentiation from drug-related tardive dyskinesia or functional movement disorders is imperative.^[22,23]^ The patient did not have any prior medical history, including a history of psychiatric medication. Although observation over a sufficient period without the patient’s attention was impossible, there were no signs of distractibility or entrainment during other motor tasks or neurological evaluations. Characteristics suggesting psychogenic dystonia, such as inconsistent movements, spontaneous remissions, movement decrease or disappearance, placebo response, false weakness, and sensory complaints, were not observed in this case.

Task-specific dystonia in focal facial muscles is a rare movement disorder that can be considered an atypical manifestation of COVID-19. This case is the first reported instance of FTSD following COVID-19 infection, which was effectively treated with botulinum toxin injections.

## Author contributions

**Supervision:** Jong-Mok Lee, Pan-Woo Ko.

**Writing—original draft:** Jong-Mok Lee, Pan-Woo Ko.

**Data curation:** Pan-Woo Ko.

**Investigation:** Pan-Woo Ko.

**Writing—review & editing:** Pan-Woo Ko.

## Supplementary Material


